# Climbing Fiber Burst Size and Olivary Sub-threshold Oscillations in a Network Setting

**DOI:** 10.1371/journal.pcbi.1002814

**Published:** 2012-12-13

**Authors:** Jornt R. De Gruijl, Paolo Bazzigaluppi, Marcel T. G. de Jeu, Chris I. De Zeeuw

**Affiliations:** 1 Netherlands Institute for Neuroscience (NIN), Royal Netherlands Academy of Arts and Sciences (KNAW), Amsterdam, The Netherlands; 2 Department of Neuroscience, Erasmus MC, Rotterdam, The Netherlands; University of Oxford, United Kingdom

## Abstract

The inferior olivary nucleus provides one of the two main inputs to the cerebellum: the so-called climbing fibers. Activation of climbing fibers is generally believed to be related to timing of motor commands and/or motor learning. Climbing fiber spikes lead to large all-or-none action potentials in cerebellar Purkinje cells, overriding any other ongoing activity and silencing these cells for a brief period of time afterwards. Empirical evidence shows that the climbing fiber can transmit a short burst of spikes as a result of an olivary cell somatic spike, potentially increasing the information being transferred to the cerebellum per climbing fiber activation. Previously reported results from *in vitro* studies suggested that the information encoded in the climbing fiber burst is related to the occurrence of the spike relative to the ongoing sub-threshold membrane potential oscillation of the olivary cell, i.e. that the phase of the oscillation is reflected in the size of the climbing fiber burst. We used a detailed three-compartmental model of an inferior olivary cell to further investigate the possible factors determining the size of the climbing fiber burst. Our findings suggest that the phase-dependency of the burst size is present but limited and that charge flow between soma and dendrite is a major determinant of the climbing fiber burst. From our findings it follows that phenomena such as cell ensemble synchrony can have a big effect on the climbing fiber burst size through dendrodendritic gap-junctional coupling between olivary cells.

## Author Summary

The inferior olive is a nucleus in the brain stem with neurons that exhibit continuous sub-threshold activity and are electrically coupled by gap junctions. It is implicated in execution and learning of motor skills and it is often assumed that it provides a teacher signal to the cerebellum. Models based on this theory generally require a continuously updated quantitative value to be sent to the cerebellum, yet the inferior olive fires spikes at a low rate of approximately 1 Hz, making reconciliation of model and biological system problematic. However, it has also been shown that olivary cells can generate an axonal burst of spikes for every somatically recorded action potential, theoretically rendering them capable of transmitting more information per event. Using a detailed neuronal model of an inferior olive cell, we examined what factors may underlie the axonal burst size. We found that leak currents between dendrite and soma and electrically coupled dendrites are major determinants. From our findings and current literature it follows that the inferior olive may be capable of adapting the speed at which motor tasks in the cerebellum are learned, depending on the synchrony of sub-threshold activity in clusters of electrically coupled cells.

## Introduction

The cerebellum ensures smooth and well-timed execution of ongoing motor tasks, making use of mainly two input channels: (1) the mossy fibers (MF) carrying information from many brain regions and thought to provide contextual information e.g. regarding sensory input from different modalities as well as ongoing motor activity and proprioceptive information, and (2) the climbing fibers (CF) carrying information about sensory events, e.g. a touch sensation on a specific part of the skin [1]. MF signals are “recoded” by the numerous cerebellar granule cells, which in turn give rise to the so-called parallel fibers (PF). In the cerebellar cortex, the PFs and CFs converge on Purkinje cells (PC). A PC receives a plethora of PF inputs, which modulate the PC's intrinsic rapid spiking behavior, and only one CF input. However, when a CF action potential (AP) is fired, the ongoing PC rapid spiking behavior is interrupted as it generates a so-called complex spike (CS) after which the PC falls silent for approximately 15 ms [2].

The inferior olivary nucleus (IO), located in the ventral brainstem, gives rise to the CFs and thus plays a vital role in the functioning of cerebellum, as indicated both by studies at the behavioral level where IO lesions or otherwise disrupted connectivity between the cerebellum and the IO lead to motor problems such as nystagmus, ataxia and dystonia [3]–[5], and by studies at the cellular level where electrophysiological data show the dramatic impact of IO activity on PC functioning [2],[6]–[8].

Because of its clear cellular structure and the impact it has on motor control, the cerebellum has inspired a number of machine learning approaches for motor control by means of acquisition of a forward or inverse model [9]–[12]. Such computational models often require a continuously updated quantitative estimate of the error in motor output from a module corresponding with the IO. Interestingly, IO cells exhibit a low firing rate of around 1 Hz under natural conditions [13]–[15], likely rendering time scales for spike coding over multiple events too long to be effective, thus decreasing the likelihood of many machine learning approaches to be an accurate representation of the cerebellar system. To better understand what information the IO can and cannot transfer to the cerebellum, a more in-depth investigation of its electrophysiological properties is required.

The IO is unique in that IO neurons are known to exhibit a slow sub-threshold oscillation (STO) of the membrane potential with a frequency that typically ranges from 2 to 10 Hz [16],[17] and its cells are densely coupled by connexin36 gap junctions [18],[19]. The timing of spiking activity is intimately tied to the STOs, occurring only within a certain phase range [7],[17]. Olivary spikes have a characteristic shape, starting with a fast sodium spike followed by an after-depolarization (ADP) due to dendritic P/Q calcium channels [20] and a long after-hyperpolarization (AHP) [13],[14]. It has been shown that olivary cells, while firing at a very low frequency, can transmit a burst of spikes along the axon for each somatic action potential [21]–[25], the spikes of which correspond with small spikelets on top of the ADP of the somatic spike [26]. It has been shown using *in silico* experiments that the shape of the olivary somatic AP may vary depending on such factors as input patterns [27]. The actual number of spikes per CF burst can vary within cells, potentially allowing for a range of values for events to be transmitted to the cerebellum via the CFs. Mathy et al. have recently shown that the CF burst size may be related to the phase of the IO cell's STO at the time the cell fired [26]. We investigated what factors primarily determine the CF burst size and the information it potentially carries by using a computational modeling approach.

## Results

### Cell model's properties

**Figure 1:**
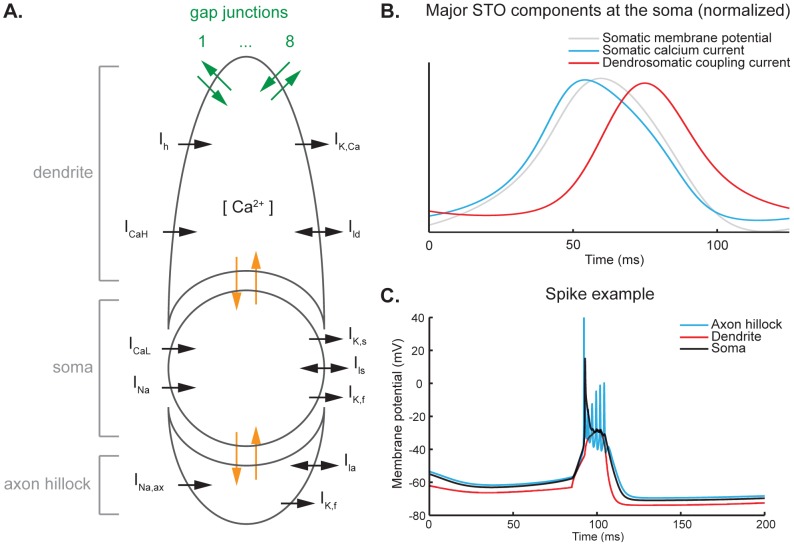
Architecture and electrophysiological properties of the cell model. **A.** Schematic representation of the three-compartmental cell model used. From top to bottom, the compartments represent the dendrite, the soma and the axon hillock (also indicated in gray on the left). Current flows internally between the dendritic and the somatic compartment as well as between the somatic compartment and the axon hillock, as indicated in orange. In addition, current can flow between a cell and up to eight cells it is connected to through the gap junctions in the dendritic compartment, indicated in green. Each compartment has its own set of ion channels. The dendrite has a high-threshold calcium current I_CaH_ (P/Q-type) and resultant internal calcium concentration [Ca^2+^], a calcium-dependent potassium current I_K,Ca_, a cationic current I_h_ and a passive leak current I_ld_. At the soma, there is a low-threshold calcium current I_CaL_ (T-type), a fast sodium current I_Na_, a potassium current with a slow component I_K,s_ and a fast component I_K,f_, and a passive leak current I_ls_. The axon hillock compartment has a fast sodium current I_Na,ax_, a fast potassium current I_K,f_ and a passive leak current I_la_. **B.** Normalized representation of the major STO components at the soma. The gray line shows the somatic membrane potential as a reference. The upward slope of the STO is caused by an activation of low-threshold calcium ion channels, leading to a depolarizing current (blue line). As the membrane potential becomes more depolarized, the calcium ion channels inactivate and current leaking from soma to dendrite increases in intensity (red line), causing the membrane potential to drop again. **C.** Example of a spike. A depolarizing current is applied at the dendritic compartment (red line), which exhibits a slow depolarization. The somatic compartment (black line) responds to this with a slow depolarization on top of which a fast sodium spike is generated. The axon hillock (blue line) shows fast sodium responses to the depolarization in the somatic compartment: the peak of the first sodium spike occurs before the somatic sodium spike (as reported by Mathy et al. [27]) and a burst of spikes is generated riding on the somatic depolarization. This burst of spikes is propagated back to the soma to some extent and is visible as spikelets on the calcium depolarization.

Our model is based largely on earlier work [7],[27],[28]. We added a compartment to model the axon hillock of the cell and enable the model to generate axonal bursts of sodium spikes. Furthermore, the somatic compartment's sodium and potassium currents were reworked, as the original model by Schweighofer et al. used an artificial sodium current to prevent generation of sodium spike bursts at the soma [28]. A schematic depicting the cell model's construction and currents is shown in Fig. 1A. The model now includes: in the dendrite a high-threshold calcium current, h current and calcium-dependent potassium current; in the soma a low-threshold calcium current, a potassium current and sodium current; in the axon hillock, a sodium current and a potassium current. All compartments also have a passive leak current and the dendritic compartment is connected to up to eight neighboring cells by means of gap junctions. A complete description of the model is provided in the supplementary Text S1.

Our model shows spontaneous and sustained STOs in a range of 6–8 Hz with an average (default) amplitude of 6.4 mV (i.e. approximately 13 mV difference measured from trough to peak), but capable of reaching STO amplitudes up to 10 mV, depending on the T-type calcium conductance value. In literature, reported frequencies for olivary STOs range from 2 to 10 Hz [14]–[17],[29] with STO amplitudes up to 10 mV occurring spontaneously [17],[29],[30]. In Table 1, we have summarized electrophysiological properties of guinea pig [13],[14],[29] as well as murine IO neurons (*n* = 6) that were observed *in vitro* to provide a clear comparison with our computational model's properties. As can be seen in Table 1, the model corresponds well with its biological counterparts, either matching them closely or falling in the range between guinea pig and mouse data. This is to be expected as the model is based on ion channel properties from multiple animals [28] (see also: supplementary Text S1). It is readily apparent that our model falls well within physiological bounds, but is a generalized IO cell model rather than a model for IO cells of a specific animal.

**Table 1:** Comparison of model properties to *in vitro* data.

	Model	Guinea pig [13] [14] [29]	Mouse (n = 6)
**STO frequency**	6–8 Hz	4–10 Hz	3–10 Hz (6±2 Hz)
**STO amplitude**	1–10 mV (default: 6.4 mV)^a^	5–10 mV	2–6 mV (5±3 mV)
**Spike ADP amplitude**	28±3 mV (20–32 mV)	49±8 mV	30±5 mV (23–38 mV)
**Spike ADP duration**	10–16 ms	20–25 ms^b^	6–12 ms (7±0.4 ms)
**Spike AHP duration**	139–241 ms (191±31 ms)	Up to 250 ms	90–165 ms (125.4±30 ms)

a Intrinsic STO amplitude based on variable T-type calcium conductance value ranging from 0.55 mS/cm^2^ to 0.9 mS/cm^2^. Default conductance value was 0.7 mS/cm^2^.

b Total calcium depolarization, including time prior to and during primary sodium spike. The actual spike ADP duration would thus be shorter.

Model properties compared to results from experiments conducted *in vitro* on guinea pig slice preparations as found in literature and from *in vitro* experiments conducted in our lab on mouse slice preparations. All values are either the observed range of values or the mean ± SD. The model generalizes the properties of IO neurons well.

To our knowledge, previous olivary cell models did not account for bursts of spikes transmitted by the axon. With the addition of the axon hillock, a burst of spikes is generated at the axon when the cell fires an action potential upon dendritic stimulation. The somatic compartment still shows only one sodium spike. The first sodium spike at the axon hillock precedes the somatic sodium spike, as has been reported earlier [26]. The spike shape in the somatic trace shows small high-frequency depolarizations on top of the ADP (Fig. 1C). These AP spikelets are the result of spikes generated by the axon hillock that are propagated back to the soma.

The olivary cell's STO is the result of interplay between ion channels in the soma and the dendrite, where differences in time constants between currents lead to oscillations rather than a stable resting membrane potential. The ion channels involved include dendritic high-threshold Ca^2+^, somatic low-threshold Ca^2+^, dendritic Ca^2+^-activated K^+^, somatic voltage-dependent K^+^ and a dendritic hyperpolarization-activated cationic current (I_h_) [14],[15],[29],[31],[32]. The principal currents that cause the oscillation measured at the soma are the somatic low-threshold Ca^2+^ current and the dendrosomatic coupling current between soma and dendrite (Fig. 1B).

IO cells are known to have firing windows outside of which spikes cannot be elicited [17]. These firing windows span approximately 180 degrees centered slightly before the peak of the oscillation, generally leading to the assumption that the peaks of the oscillation are closer to the firing threshold. However, this explanation fails to account for often described skewed distributions as it assumes a constant firing threshold, which is not biologically plausible [33]. Our model shows a firing window similar in size to that of its biological counterpart, albeit shifted towards the upward slope of the oscillation by approximately 45 degrees. The upward slope of the STO is generated mostly by calcium currents, facilitating depolarization of the membrane by excitatory input. At the peak of the oscillation, calcium channels inactivate while potassium channels are activated and the dendrosomatic coupling current increases, bringing the membrane potential back down, but also making the cell much harder to excite (Fig. 1B). As a result, our model's firing window ranges from approximately 1.6π to a little over 0.5π and provides an explanation in terms of ion flux for the firing window reported in previous work [17].

### Phase-dependency of burst size

To assess whether the generation of the climbing fiber burst is dependent on the phase of the STO, we ran simulations mimicking the experiment done by Mathy et al. in which a 5 Hz sinusoidal sub-threshold oscillation of the membrane potential was imposed on the cell by injecting a corresponding sinusoidal current [26]. In addition, we simulated an equal number of control experiments where the cell received no such current injection and would thus follow its intrinsic STO. Since Mathy et al. stimulated fiber beams to provide input to the cell they recorded from, it is hard to tell whether either clusters of coupled cells or single cells were stimulated. Therefore, we ran the simulations under two conditions: one in which the entire network was stimulated at once and the other in which only the recorded cell received excitatory input.

**Figure 2:**
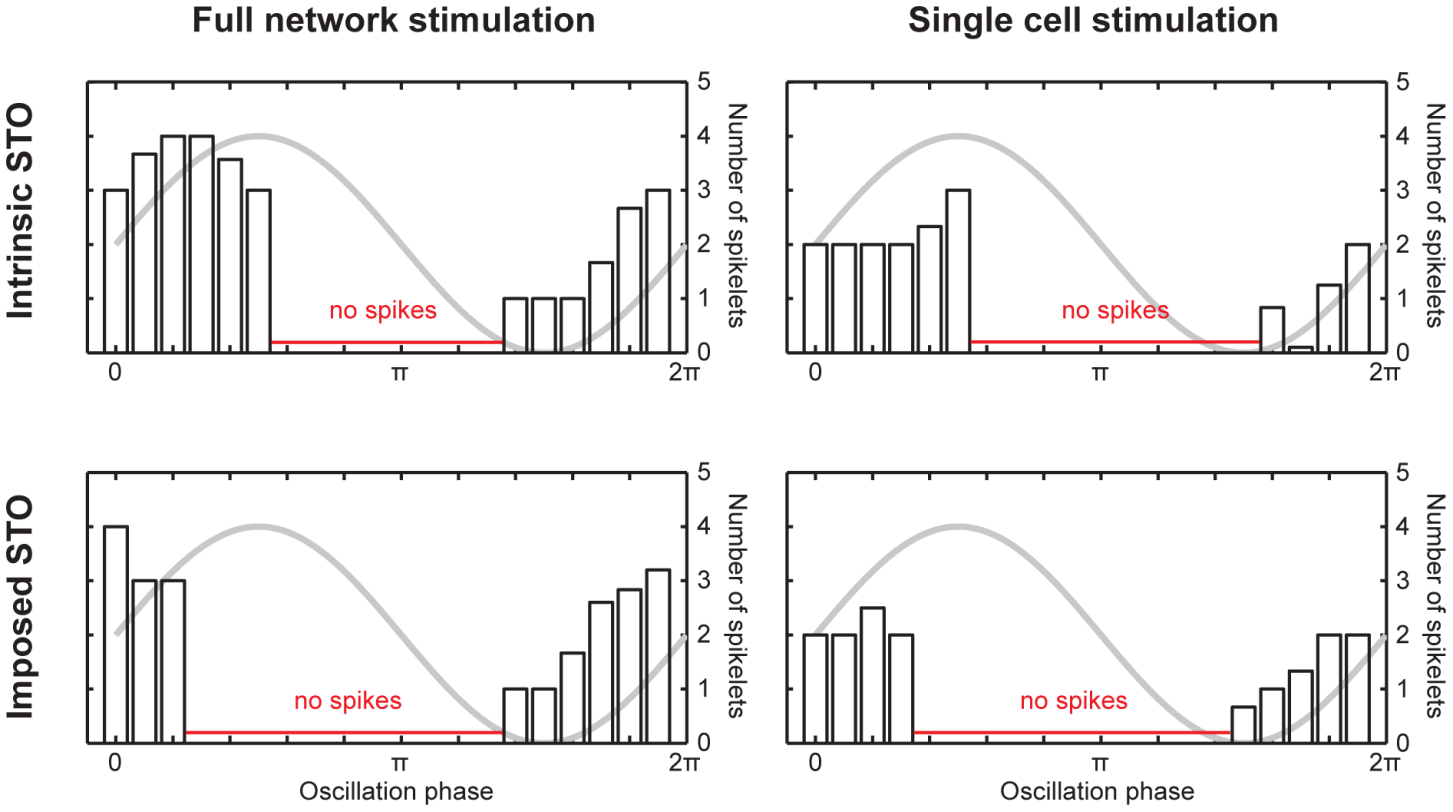
Phase dependency of AP spikelet counts. Phase dependency of IO cell AP spikelet count under natural (intrinsic STO) conditions and when a sinusoidal 5 Hz STO is imposed through current injection. Left panels show the results for full network stimulation, corresponding with e.g. stimulating a fiber beam, whereas the right panels show the results for single-cell stimulation. Under all four conditions, the STO establishes a firing window outside of which the cell does not fire action potentials (the phase range where spikes were not generated is indicated in red), in concordance with earlier findings [17]. However, the actual boundaries of the firing window are different when an oscillation that differs from the intrinsic STO is imposed (bottom panels as opposed to top panels). When the entire network is stimulated, there is a clear phase-dependency of AP spikelet count, as the spikelet count ranges from 1 to 4 depending on the phase (left panels). However, when only one cell in a cluster of 9 cells is stimulated, this phase dependency is less clear, as the spikelet count can still take different values, but is of limited variability and generally equals 2 (right panels).

As can be seen in Fig. 2, there are differences in firing behavior between cells that are following an intrinsic or an imposed STO, but there appears to be a phase dependency for the burst size in both cases. The different results between the STO types are due to the dynamics of the ion channels involved, which are voltage-gated. The membrane potential of the imposed STO changes more slowly than it would compared to that of the cell's intrinsic STO, due to the lower frequency of the imposed STO. This allows for increased activation of the potassium currents at the peaks of the oscillation and increased inactivation at the troughs. This leads to a slight phase shift of the firing window as well as states of activation and inactivation of the calcium and potassium channels that would not occur under more natural circumstances. Despite the artificial nature of the imposed STO experiments, there appears to be a phase-dependency of the number of AP spikelets as was described earlier. The outcome of experiments using an imposed STO can likely be considered valid, judging by the similar outcome predicted by the model for simulations in which only intrinsic oscillations occurred and those with imposed oscillations.

Interestingly, the phase-dependency is much more pronounced in the simulations where the entire 9-cell network was stimulated rather than just the center cell in the 3×3 grid. This is in line with the results Mathy et al. show in their supplementary material, where direct stimulation of single cells at different intrinsic STO phases yields a much less pronounced phase dependency, ranging from an average 1 to 1.3 spikes [26]. The only explanation for this difference in phase-dependency of the axonal burst size in our simulations is that the dendrodendritic gap-junctional coupling currents through connexin36 gap junctions also play a part in determining the final spike shape, as this difference in spike shape between full-network and single-cell stimulation occurs even when the network states at the time of stimulation are identical.

### ADP size, Ca^2+^ and coupling currents

**Figure 3:**
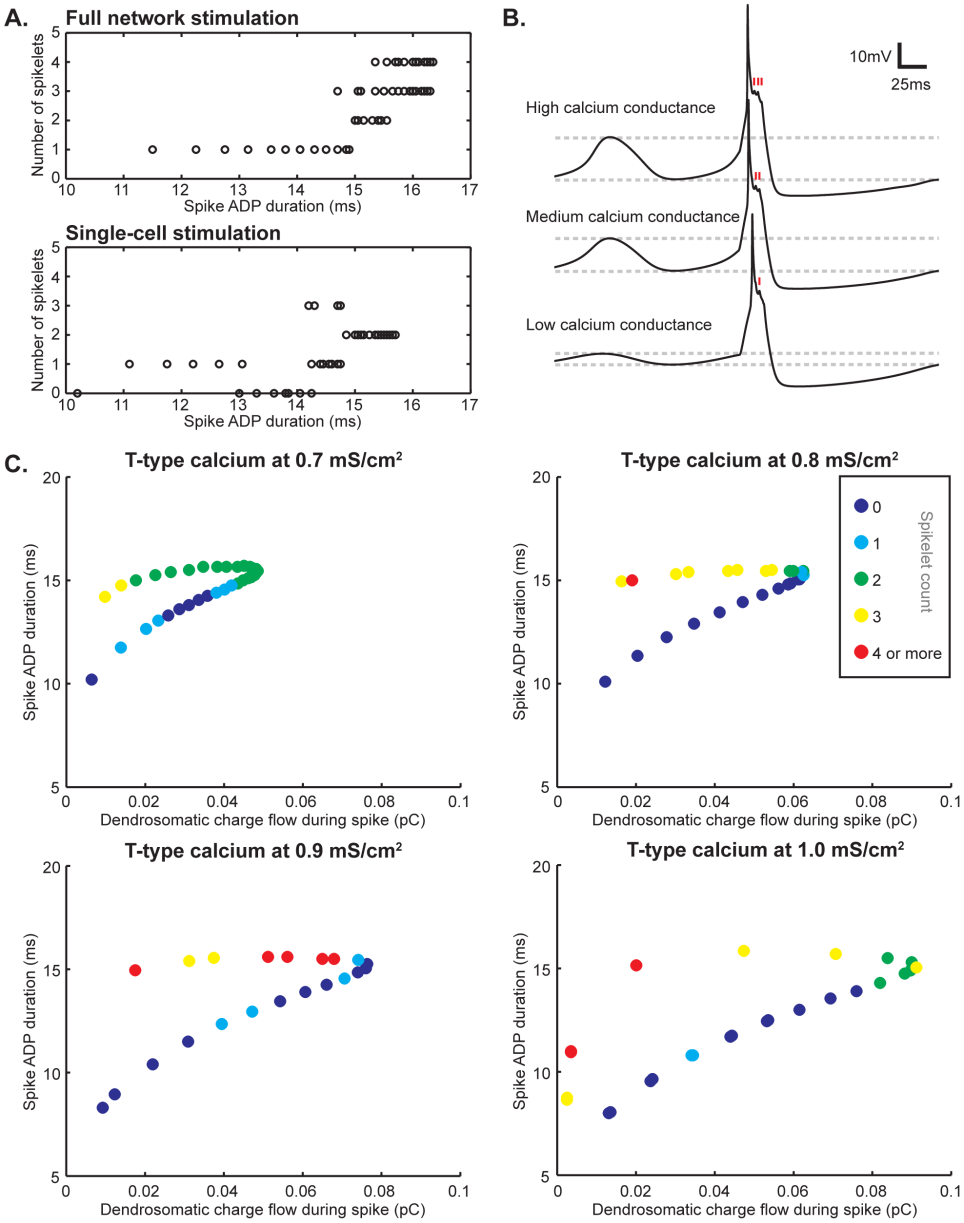
Factors underlying AP spikelet count. **A.** Correlation between spike ADP duration and AP spikelet count. In general, a longer ADP increases the chances of larger amounts of spikelets on top of the somatic ADP. This is most readily apparent when stimulating the entire network (top panel). Still, the ADP duration by itself does not provide an adequate explanation for the amount of spikelets that are part of the spike shape, since the number of spikelets can still vary considerably even within a millisecond bin both when the entire networks fires and when only one cell does. When only one cell in the network fires, the relation between ADP duration and number of spikelets does not appear to be linear, even though on average a higher number of spikelets is still more likely at longer ADP durations (bottom panel). **B.** Changing the intrinsic conductance values of the low-threshold calcium current changes the amplitude of the oscillations (as indicated by the dashed lines aligned with the peaks and troughs of the depicted traces), but also the number of spikelets (time of occurrence is indicated with red markers in each depicted trace). The number of spikelets decreases as the T-type calcium expression level decreases. **C.** Spike ADP and dendrosomatic coupling currents as AP spikelet count determinants. Single cells in a 9-cell network were stimulated for all of the simulation results shown. Warmer colors represent higher numbers of AP spikelets (color-coding is the same for all four panels and indicated in the figure). Spike ADP duration and dendrosomatic charge flow form a trajectory. It is readily apparent from all four panels that higher numbers of spikelets are more likely to occur at longer ADPs, but in addition decreased charge flow increases the chance of generating an AP with more spikelets. As a result, the prediction of AP spikelet count can be improved when taking both ADP duration and dendrosomatic charge flow into account. At different T-type calcium expression levels, the range of possible spikelet counts and the distributions thereof vary. Clearly, spike ADP and dendrosomatic coupling currents are major determinants for the AP spikelet count measured at the soma, but other currents both intra- and intercellular can cause local phenomena in the distributions along the trajectories shown.

Axonal burst sizes can vary depending on the phase of the oscillation, which in turn is determined by the state and availability of the cell's ion channels. We analyzed the relation between the spike's ADP duration and the number of spikelets on top of it. Indeed, with a longer ADP the chances of higher numbers of spikelets are increased, but as can be seen in Fig. 3A (most notably when only one cell fires an AP) the ADP duration alone does not provide an adequate explanation for variability in spikelet counts. In the previous section we showed that stimulating a single cell in the network yielded no clear correlation of AP spikelet counts with STO phase. Therefore, we attempted to establish a more direct link to the contribution of currents involved in the STO at the soma to the climbing fiber burst size.

Since T-type calcium channels are a driving factor in IO cells' STOs [34] and these oscillations in turn are intimately tied to the firing properties of the cell by setting a firing window [17] and possibly determine the burst size transmitted through the axon [26], we investigated the electrophysiological effect of variable T-type calcium expression levels. At lower expression levels, the STO amplitude is decreased and the frequency is slightly lower due to a slower activation of potassium channels in response to the slowed and lessened calcium depolarization. Not only is the activation of potassium channels slower (both somatically and dendritically), but it also reaches lower peak values, resulting in larger or even full-phase firing windows at very low STO amplitudes.

The calcium depolarizations that are part of somatic action potentials are of a shorter and less variable duration at lower conductances and the AP spikelet counts are also much less variable. At a T-type calcium conductance value of 0.55 mS/cm^2^, our model showed no variability in spikelet counts: all APs had 1 spikelet regardless of STO phase at the time of firing. Only from a conductance value of 0.7 mS/cm^2^ and up was there a notable range of spikelet counts and ADP durations in our simulations: from 0 to 3 spikelets in the case of 0.7 mS/cm^2^, though 4 or, in exceptional cases, even up to 6 spikelets can occur at higher T-type calcium ion channel expression levels.

Even though larger somatic T-type calcium currents could potentially lead to bigger ADPs by additional activation of dendritic P/Q calcium channels, the effect on ADP duration is very small in our model when changing only the T-type calcium conductance values. We observed a somewhat increased ADP at higher T-type calcium conductance values, but most notably an increased range of current flow between soma and dendrite as well as a range of somatic ADP widths extending to shorter durations (below 10 ms), indicating increased dendritic calcium-dependent potassium activation. Upon inspection, the charge flowing from soma to dendrite combined with the ADP duration taken together form a fairly reliable indicator for a cell's spikelet counts, as these factors combined describe a trajectory along which AP spikelet distributions unfold (Fig. 3C). It is clear that even though ADP duration and dendrosomatic charge flow do not provide a full explanation for the spikelet counts, they form the main determinants for the distribution: the longer the ADP, the higher the chance of more AP spikelets and the smaller the dendrosomatic coupling current, the higher the chance of more AP spikelets.

### Olivary action potentials in heterogeneous networks

Coupling currents between soma and dendrite are a determinant of CF burst size. Gap junctions can influence these currents. Current flow through gap junctions occurs when the dendritic membrane potentials of coupled cells differ. These differences can manifest *on occasion* when coupled cells do not spike simultaneously (as has also been shown in the previous sections), but also *continuously* under circumstances where coupled cells have different intrinsic STO properties or when their oscillations are out of phase. All of these conditions are likely to be applicable under natural circumstances. This raises the question what the CF burst size variability is *in vivo* and what it might encode.

We tried to ascertain the variability of spikelet counts in a more biological setting by making the simulated networks have heterogeneous T-type calcium expression levels. Such heterogeneity leads to differences in STO frequency and amplitude between cells [35]. We used a conductance range for the low-threshold calcium current where all individual cells show intrinsic STOs (0.55 to 0.9 mS/cm^2^). In addition, we used several network sizes of 3, 9 or 25 cells as it has been shown that the number of coupled IO cells within a densely coupled cluster can vary [36]. Despite differences in intrinsic STO period and amplitude, our simulations show that a cluster of 25 coupled cells or less always settles on one stable STO period across all cells in approximately 1s (see also Fig. S1 A and B). Our observation that cell ensembles synchronize despite intrinsic STO differences is in line with theoretical work of others [35]. Before the STOs are maximally synchronized, the STO amplitude in the cells is smaller due to dendrodendritic coupling currents through the gap junctions (Fig. S1A). In the biological system, coupled IO cells are to a certain extent electrically coupled with other clusters that need not be in phase with their STOs [37] and neurons within a cluster can hypothetically receive different input patterns, causing phase shifts in the STO due to cell-intrinsic resetting properties. We investigated the effect of coupling currents due to differences in sub-threshold activity between cells on AP spikelet distributions. We used the STO amplitude as a measure of STO synchrony between cells in the network for ease of comparison with *in vitro* and *in vivo* work, as cells' STO amplitudes increase the more they synchronize their STOs with cells they are coupled to (Fig. S1).

For the phase analysis we divided the data into two sets of comparative size based on STO amplitude. The boundary at which both sets had an equal amount of data points by approximation was at 5.5 mV, corresponding with a mean network synchrony ratio of approximately 0.85 (Fig. S1C). Cells that showed STO amplitudes greater than 5.5 mV prior to stimulation had smaller dendrodendritic gap-junctional coupling currents than cells with smaller STO amplitudes and were considered to be part of a cell ensemble close to synchrony. Cells that showed STO amplitudes smaller than 5.5 mV were considered to be part of a non-synchronized network.

**Figure 4:**
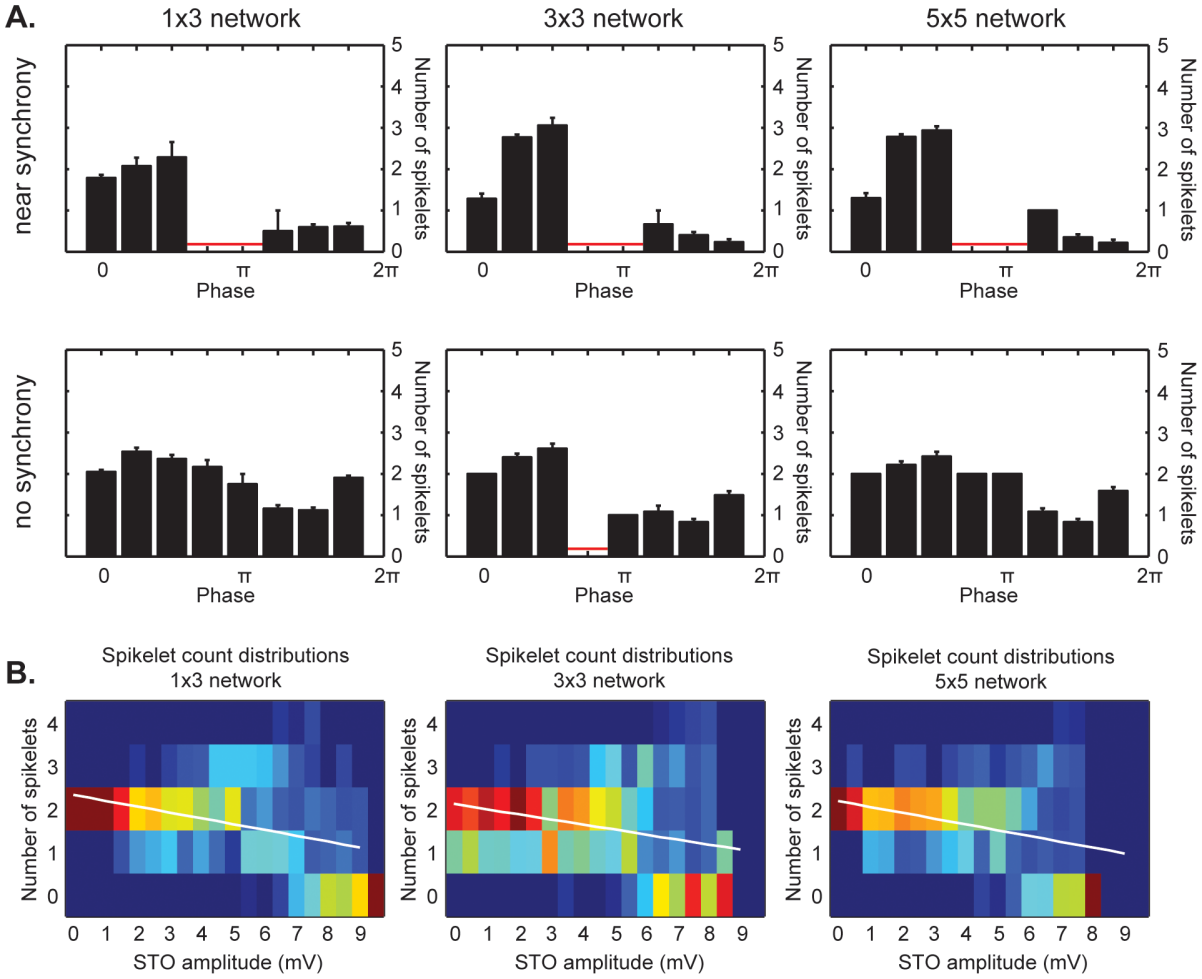
Effects of network synchrony on AP spikelet count. **A.** Phase dependency of AP spikelet count for different network sizes when a network consists of coupled cells with near-synchronized STOs (STO amplitude >5.5 mV, top row) or non-synchronized STOs (STO amplitude <5.5 mV, bottom row). Phase ranges where no spikes were fired are indicated in red. *Top row, near synchrony* - The small 1×3 network shows a weak phase dependency of the AP spikelet count, averaging 0.5 spikelets on the upward slope of the STO and 2 spikelets around the peak. The 3×3 and 5×5 networks show similar outcomes and a stronger phase-dependency of the AP spikelet count, averaging 0 spikelets on the upward slope, followed by 1 spikelet near the peak and 3 spikelets around the peak. Due to a strong depolarizing current (5 pA, 20 ms for 1×3 networks and 6 pA, 20 ms for 3×3 and 5×5 networks, see Methods), spikes are sometimes fired outside the usual firing window in the trough of the oscillation (1.25π radians bin, total of 7 occurrences in 2100 simulations across all network sizes). *Bottom row, no synchrony* - Regardless of network size, there is no clear phase-dependency of AP spikelet count. A rounded average of 2 spikelets is seen across all phases, except for part of the phase range falling outside or close to the bounds of the firing window where a rounded average of 1 spikelet may occur. Due to analysis restrictions imposed by determining the phase, STO amplitudes smaller than 1 mV are poorly represented in the data set. **B.** AP spikelet distributions across STO amplitudes for different network sizes. Warmer colors denote more occurrences, cooler colors less. The distribution of spikelets changes as a function of STO amplitude. In a range of approximately 5 to 7 mV, the distribution broadens, corresponding with the phase dependency of AP spikelet counts shown in panel A. At an STO amplitude of 7.5 mV or more, this distribution narrows again, but at a lower average spikelet count than was seen at lower amplitudes. The average number of AP spikelets shows a downward trend for all network sizes, as illustrated by the fitted trend lines shown in white.

Networks that operated near synchrony had a preferred firing window and showed some phase dependency of spikelet counts (Fig. 4A, top row). Stimulation was relatively strong, causing a few spikes outside the expected firing window phase range around 1.25π radians. Disregarding the 1.25π radians bins due to the limited number of data points (7 occurrences in 2100 simulations), there appears to be a coarse 3-value phase correlation of spikelet counts in 3×3 and 5×5 networks: 0 to 1 spikelets on the rising slope of the STO and 3 spikelets near the peak. The 1×3 networks show less of a correlation between phase and AP spikelet count in the firing window, generally generating 2 AP spikelets on the rising slope to the peak of the STO and either 0 or 1 spikelet otherwise. The observed phase dependency was strongest in the 5.5 mV to 7 mV STO amplitude range for all networks with STOs near synchrony.

For cells with STO amplitudes below 5.5 mV, and thus part of non-synchronized cell ensembles, no correlation between phase and AP spikelet count was found and no clear firing window was present, due to decreased potassium activation (Fig. 4A, bottom row). APs fired by cells under these circumstances showed 2 spikelets on average, and sometimes 1 if the AP occurred outside normal firing window. It is important to note that our phase analysis required two peaks and two troughs prior to the AP. Therefore only networks that had gotten some time to synchronize were included, causing a slight overrepresentation of higher-amplitude STOs where the firing window is more clearly present.

As the STO amplitude increases, the distributions of AP spikelet counts change and the average number of AP spikelets decreases in general (Fig. 4B). At the highest STO amplitudes of 7.5 mV and up, there is a decrease in AP spikelet occurrences, often resulting in only 0 or 1 spikelets occurring regardless of network size over an increasingly broad portion of the firing window. Networks that achieved these amplitudes in cells generally had high average T-type calcium expression levels and high levels of dendritic potassium activity as a result, terminating AP and ADPs quickly. Interestingly, cells with the lowest T-type calcium expression levels could exhibit higher AP spikelet counts (up to 3) as well as higher STO amplitudes if they were coupled to cells with higher T-type calcium channel expression levels, whereas in isolation or when coupled to identical cells the cells with a low T-type calcium expression level showed only few spikelets. Thus, gap junctions also serve as a normalizing factor for STO amplitudes and AP spikelet counts across cells.

## Discussion

Our work provides new insights in how olivary AP spikelet distributions may unfold under different conditions. Taking advantage of the control a detailed model gives, we investigated the effect of specific currents on IO neuron firing properties, while also taking into account the effects thereof on parameters available in empirical data, such as AP spikelet count and STO amplitude.

Our three-compartmental model faithfully reproduces earlier findings on olivary neurons such as the frequency and shape of their STOs, their propensity to limit APs to a preferred firing window, and their spike shape as recorded at the soma using whole-cell patch-clamp techniques. There appears to be a difference in the phase range of the firing window reported in earlier work from our lab [17] in that the firing window is phase-shifted. However, since the actual shape of the oscillation can vary (i.e. is not truly sinusoidal nor identical between cells), the phase offset of the best sinewave fit can vary and even relative phase differences within a trace are suspect. In most oscillation shapes in our data there is a slightly steeper inclination of the upward slope (see Fig. 1B and Fig. S1A) and a shorter duration of the peak of the STO relative to the trough (see Fig. 3B and Fig. S1A). Because of this, spikes appear to fall in a relatively small phase range of the peak since the actual peak of the STO is shorter than that of the fitted sinewave. Due to the difficulty in fitting sinewave functions to STOs, spikes mapped to the fitted sinewave by our automated analysis software could be considered to be phase-shifted with respect to the actual data when judged by a human: the spikes fall almost exclusively on the rising slope of the fitted sinewave, but start after the trough minimum and extend beyond the peak of the STO in the actual data. In addition, the results from our lab regarding the occurrence of a spike relative to STO phase were generally obtained by fitting a sinewave to the data, but relying on user input for assigning the event to one of eight 45-degree bins, thus potentially introducing a slight bias and explaining the discrepancy between previously reported firing windows and that of our model, at least in part.

In a network setting, our model shows a clear phase-dependency of AP spikelet count in line with findings by Mathy et al. [26] when stimulating the entire network of coupled cells. When stimulating only one cell in the network this phase-dependency is largely lost, again in line with the results shown by Mathy et al. [26]. Thus, our model not only reaffirms previous findings, but also provides an explanation for seemingly contradicting results. However, there are also differences. Mathy et al. reported a maximum AP spikelet count at ∼π radians, which does not match the maximum we found in simulations similar to their experiment (∼0.5π). We speculate that this is due to a difference in the mapping of STO phase. Furthermore, their results span the full range of phases of the STO, indicating that in their experiments spikes could be elicited regardless of STO phase.

Calcium currents are the cause of the IO cells' typical spike ADP and low-threshold voltage-dependent calcium ion channels have been shown to be important in the generation of the IO cells' STOs [20],[34]. Indeed, with variable somatic low-threshold calcium ion channel expression levels our model shows substantial changes in the STO amplitude and smaller changes in the STO period and in the ADP size. The small change in ADP size was the result of increased dendrosomatic coupling currents at higher calcium ion channel activation levels. Our model predicts that the duration of the ADP and the amount of current flow between the soma and the dendrite are the main determinants of the somatic AP spikelet count.

Since coupling currents between soma and dendrite are a major AP spikelet count determinant, gap-junctional coupling plays a role in establishing the CF burst size. Furthermore, the different CF burst size results between full-network and single-cell stimulation in our simulations also point to an effect of gap junctions on AP spikelet count. The reason for this is that ensemble firing minimizes membrane potential differences between cells, causing less dendrodendritic gap-junctional coupling currents to affect the cells' internal dendrosomatic coupling currents.

Single cells firing due to dendritic stimulation in a more biologically plausible setting with variable low-threshold calcium ion channel expression levels among cells and different coupled cell ensemble sizes show higher average AP spikelet counts and no phase-dependency of the CF burst size at low STO amplitudes. In a certain range of STO amplitudes, there appears to be a relation between STO phase and spikelet count in what might be a coarse coding, but at other STO amplitudes, both larger and smaller, the phase-dependency of climbing fiber burst size shown earlier using *in vitro* preparations may be lost. The artificial current injection used in those experiments could impose an oscillation on a cell ensemble and thus create artificial synchrony at moderate STO amplitudes. Under these conditions, our model predicts a correlation between STO phase and AP spikelet count in line with the findings by Mathy et al. [26].

A quantitative error estimate is required in several computational cerebellar models for learning, but so far these have been hard to explain using IO data due to the low firing rate of IO cells. It has been proposed that variability in spike shape of olivary cells may allow for transmission of more information per event than would otherwise be possible [27]. Phase dependency of the CF burst size and somatic AP spikelet count would allow for a coarse, low-resolution encoding of temporal information. Our model predicts that there may be a phase-dependency of climbing fiber burst size, but only in a limited range of STO amplitudes and in coupled cell ensembles of sufficient size.

We provide as an additional hypothesis that IO ensemble synchrony, for which STO amplitude could be used as an indirect measure, modulates the CF burst and in turn allows for online adaptation of learning speeds. Our model predicts that, as synchrony increases, STO amplitude goes up and the average number of AP spikelets goes down. The PF/PC synapse is modulated by CF activity and the size of the CF spike burst may determine the plasticity effect [26],[38]. Keeping this in mind, CF burst size could well be an indicator of the state a learning process is in, allowing quick and coarse learning at low ensemble STO amplitudes (i.e. low synchrony) and then more refined learning at higher amplitudes. Since single CF spikes have been shown to cause LTP in PCs rather than LTD [26],[38], the highest STO amplitudes where few AP spikelets occur are conceivably a way of unlearning (preventing the biological equivalent of overfitting data) or converting the direction of learning. A system that has a way of adequately adapting the coarseness with which it learns online can speed up a learning process without sacrificing precision.

It is important to note that simulation results never translate directly to empirical data: our model utilizes ion channel descriptions taken from *in vitro* preparations from several animal species. It demonstrates principles underlying the generation of olivary STOs and spikes, but such things as the actual number of AP spikelets or the time between two such spikelets may differ from data acquired from an actual cell, much as it may also differ between species. Even so, our model provides valuable insight into how olivary AP spikelet distributions may unfold under different conditions and even how meaningful the CF burst may be *in vivo*.

## Methods

### Ethics statement

As required by Dutch legislation, the experiments were approved by the institutional animal welfare committee (DEC, Erasmus MC, Rotterdam, The Netherlands).

### *In vitro* electrophysiology and data analysis

Coronal slices of the brainstem of 3- to 6-week-old mice (adult) were kept in ACSF containing (in mM): 124 NaCl, 5 KCl, 1.25 Na2HPO4, 2 MgSO4, 2 CaCl2, 26 NaHCO3 and 20 D-glucose, bubbled with 95% O2 and 5% CO2 (all chemicals were purchased from Sigma-Aldrich). Whole-cell patch-clamp recordings were performed at physiological temperatures (34–35°C) using an EPC-10 amplifier (HEKA Electronics, Germany). The patch pipettes were filled with intracellular solution containing (in mM): 120 K-gluconate, 9 KCl, 10 KOH, 3.48 MgCl2, 4 NaCl, 10 HEPES, 4 Na2ATP, 0.4 Na3GTP, and 17.5 sucrose; pH was adjusted to 7.25. Input resistance (Ri) was measured by injection of hyperpolarizing test currents (200 pA; 100 ms) and was calculated from the voltage transient toward the end of current injection. Recordings were excluded if the input resistance varied by >15%. Data was analyzed in Clampfit 9.2 (Axon Instruments, Foster City, CA) and in Matlab (Mathworks).

STO frequency was estimated with a custom-made routine based on a fast-Fourier transform function, STO amplitude was averaged over the peaks and the troughs of multiple oscillations. The AP onset is measured as the time point on the sinusoidal oscillation where the second derivative (with respect to time) of the voltage trace is maximal. From that time point ADP height was calculated. The ADP duration is measured over the portion of the spike after the primary sodium spike until the membrane potential reaches the value attained at the peak of the STO. AHP length was calculated from the end of the ADP until the time point at which membrane potential reached the level preceding the AP.

### Cell model

We ran simulations using a three-compartmental model based on existing two-compartmental models [7],[27],[28]. In the current model, somatic sodium and potassium currents are revised and a compartment representing the axon hillock has been added. The axon hillock compartment has voltage-dependent sodium and potassium channels and a passive leak current. Maximum coupling strength between olivary cells was uniformly set at 0.04 mS/cm^2^ for all simulations of ensembles of cells. When cell ensembles were simulated, the cells were spatially arranged on a grid and the center cell of the network was used for analysis. A detailed description of the model is provided in the supplementary Text S1.

The number of spikelets was determined at the soma rather than the axon hillock compartment. This allowed us to make a direct comparison between olivary spike shapes in literature and data from our own lab as a quality control. Furthermore, since most recordings are performed somatically it facilitates direct comparison between our model's predictions and lab work. A spikelet was defined as a local maximum in the membrane potential over the span of the calcium depolarization following the initial somatic sodium spike. The phase of a spike was determined by fitting a sinewave to two periods of the oscillation directly preceding the spike and determining the phase of the sodium spike maximum relative to that fit. Since the STO peaks and troughs are not symmetrical, we used a second-order polynomial fit to estimate the location of the peak and trough maxima to which potential sinewave fits were aligned. After determining the locations of peak and trough maxima, the best fit aligned to either two minima or two maxima was selected. The STO amplitude estimate was based on the minimum and maximum of the STO period directly preceding the spike.

### Phase-dependence simulations

The phase-dependency experiment simulations were run using 3×3 coupled networks. Cells were connected to their directly neighboring cells (up to eight) without thoroidal connections. The center cell was either oscillating at 5 Hz due to an imposed sinusoidal membrane potential or allowed to follow its intrinsic oscillation. In either case, 100 simulations were run with excitation by means of a dendritically applied current (20 ms block pulse, 5.5 pA) at different times (2 ms later for each simulation in the sequence). The imposed 5 Hz sinusoidal oscillation used had the same maximum and minimum membrane potential values as the cell's intrinsic oscillation.

### Ensemble synchrony simulations

We ran simulations for different neuron cluster sizes. Cells were arranged in a grid and connected to their directly neighboring cells (up to eight). The grid sizes used for these simulations were: 1×3, 3×3 and 5×5 cells.

Networks were initialized with random phase differences between cells and with variable T-type calcium conductance values ranging from 0.55 mS/cm^2^ to 0.9 mS/cm^2^ keeping the average around 0.7 mS/cm^2^. During a simulation, the center cell in the grid received a depolarizing current (6 pA) applied dendritically for 20 ms starting at a random point in time between 200 ms and 900 ms into the simulation, where approximately 1000 ms is typically the longest time needed for a randomly initialized network to become fully synchronized. The chosen depolarizing current is relatively high, giving rise to action potentials in a broader range of phases. For the 1×3 networks, a slightly lower depolarizing current was used (5 pA) in order to avoid over-stimulating the cell due to reduced current flow to neighboring cells while keeping the chance of generating action potentials due to stimulation similar (69% for 1×3 networks as opposed to 66% for larger networks). For every network size, a total of 700 simulations was run and trials with a full-blown action potential were analyzed for timing of the spike w.r.t. the phase of the STO, the number of wavelets per spike and the STO amplitude.

## Supporting Information

Figure S1 **Synchronization properties of 3×3 and 5×5 networks. A.** An example of somatic membrane potential traces during synchronization in a 3×3 simulated network of IO cells with different STO properties due to variable T-type calcium expression levels. The network was initialized with random phase differences. At the start, STO amplitudes are small and phase and frequency differences are apparent. The coupled cells quickly settle into one rhythm, but due to phase differences of active currents between cells, STO amplitudes are small initially. Note that the magnitude of cells' STO phase differences are thus indirectly represented by the amount by which STO amplitudes are decreased. As time goes by, the cells synchronize their active currents, thereby maximizing the STO amplitude. The maximum amplitude of individual cells remains variable, due to the heterogeneity of the T-type calcium expression levels. **B.** Synchrony over time as decreasing mean phase difference and increasing synchrony ratio for both 3×3 (left panel) and 5×5 networks (right panel), *n* = 250 for both graphs. Plots are mean ± SD. Phase difference in degrees was determined at t = 0 and approximated over time by comparison to maximum attained mean STO amplitude over the total runtime of the simulation. The maximum attainable phase difference is 180° (counterphase), which was considered a synchrony ratio of 0, whereas synchrony was maximal when the phase difference was 0°. As can be deduced from the plots, near-perfect synchrony was generally attained well within 1000 ms, with most of the synchronization occurring in the first 500 ms of the simulated network activity. **C.** Network synchrony statistics when crossing the STO amplitude threshold set at 5.5 mV for both 3×3 and 5×5 networks. Bar plots show mean value ± SEM and *n* = 250 for both network sizes. Time until the 5.5 mV STO amplitude threshold is crossed for 3×3 networks is 241±11 ms on average and 308±10 ms on average for 5×5 networks (left panel). The mean phase difference and synchrony ratio of the network at the time the 5.5 mV STO amplitude threshold is crossed is 26.7±1.1° and 0.85±0.006 synchrony for 3×3 networks and 21.1±0.8° and 0.88±0.005 synchrony for 5×5 networks.
(PDF)

Text S1 This supplementary text contains a full description of the dendritic, somatic and axon hillock compartments of the computational model used.
(PDF)
