# Supplementary figures and images for: Climbing Fiber Burst Size and Olivary Sub-threshold Oscillations in a Network Setting
Source: PLoS Comput Biol. 2012 Dec 13;8(12):e1002814. doi: 10.1371/journal.pcbi.1002814 (PMC3521668; doi:10.1371/journal.pcbi.1002814)

**A.**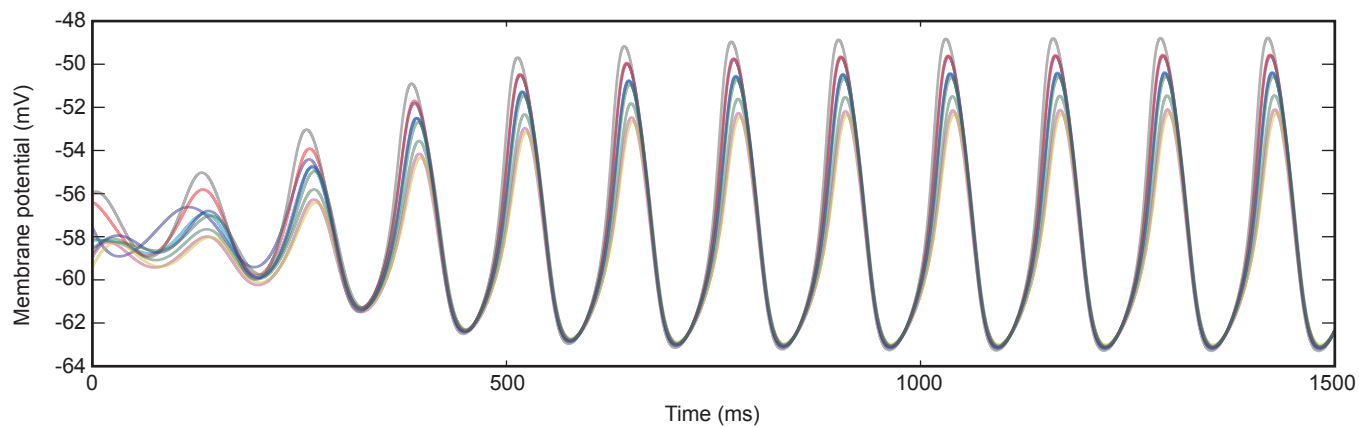**B.**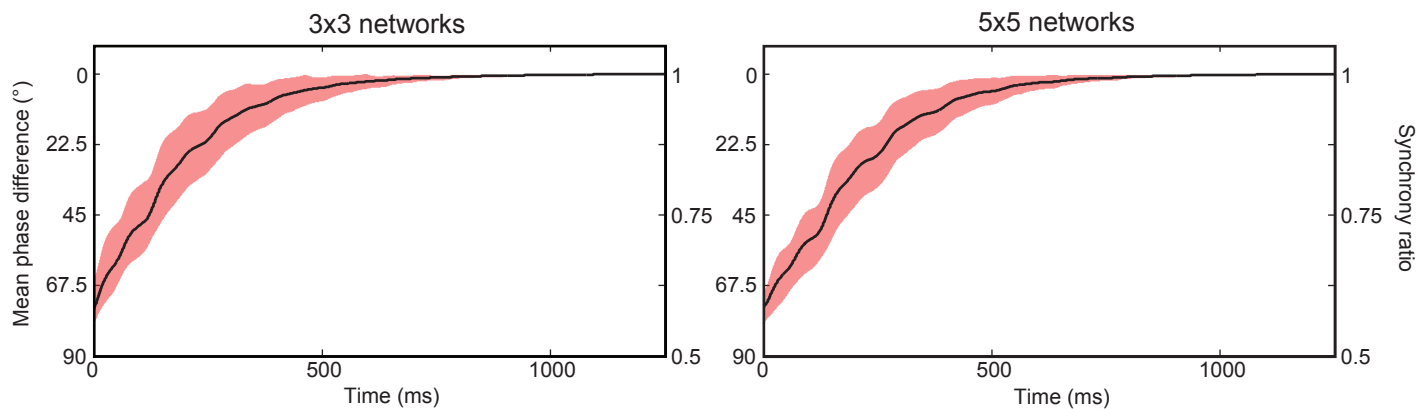**C.**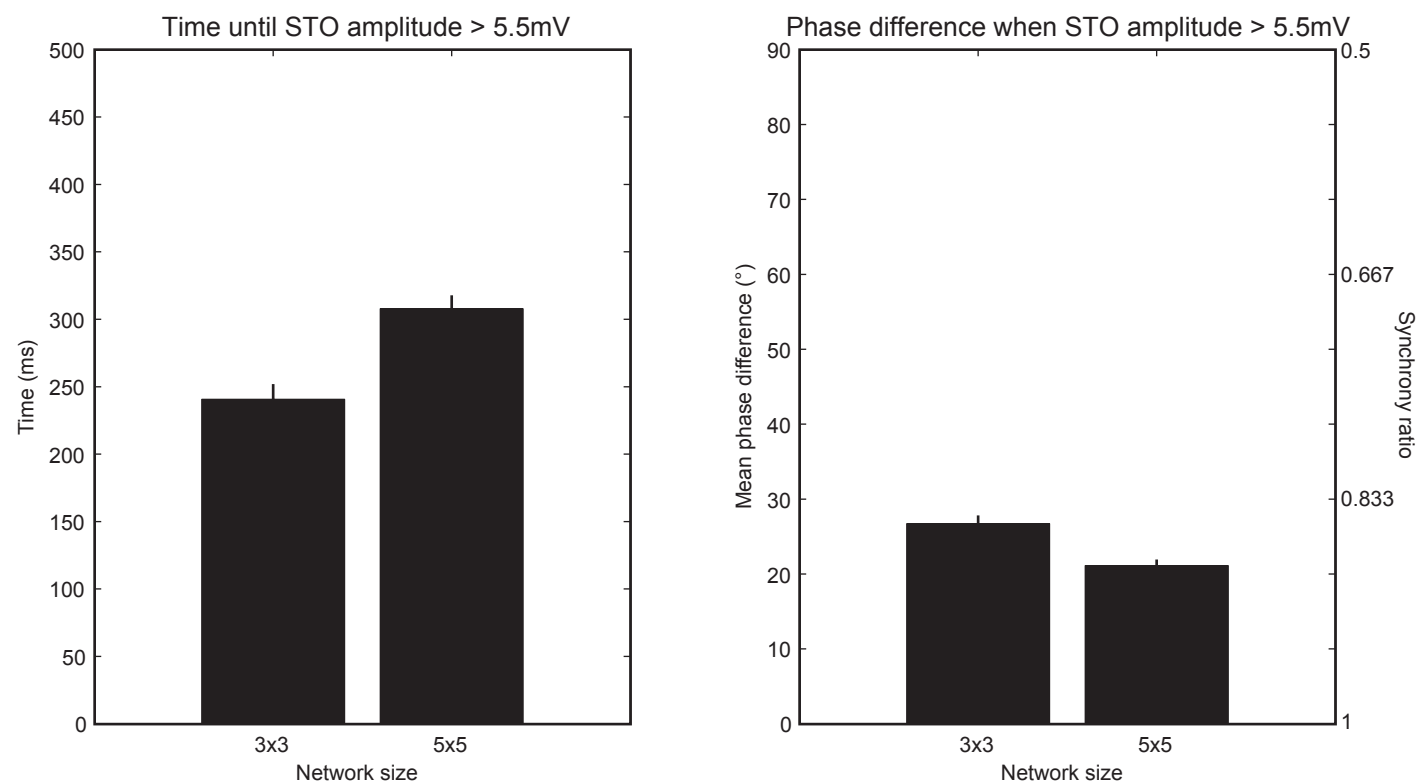

Supplement: Figure S1 — Synchronization properties of 3×3 and 5×5 networks. A. An example of somatic membrane potential traces during synchronization in a 3×3 simulated network of IO cells with different STO properties due to variable T-type calcium expression levels. The network was initialized with random phase differences. At the start, STO amplitudes are small and phase and frequency differences are apparent. The coupled cells quickly settle into one rhythm, but due to phase differences of active currents between cells, STO amplitudes are small initially. Note that the magnitude of cells' STO phase differences are thus indirectly represented by the amount by which STO amplitudes are decreased. As time goes by, the cells synchronize their active currents, thereby maximizing the STO amplitude. The maximum amplitude of individual cells remains variable, due to the heterogeneity of the T-type calcium expression levels. B. Synchrony over time as decreasing mean phase difference and increasing synchrony ratio for both 3×3 (left panel) and 5×5 networks (right panel), n = 250 for both graphs. Plots are mean ± SD. Phase difference in degrees was determined at t = 0 and approximated over time by comparison to maximum attained mean STO amplitude over the total runtime of the simulation. The maximum attainable phase difference is 180° (counterphase), which was considered a synchrony ratio of 0, whereas synchrony was maximal when the phase difference was 0°. As can be deduced from the plots, near-perfect synchrony was generally attained well within 1000 ms, with most of the synchronization occurring in the first 500 ms of the simulated network activity. C. Network synchrony statistics when crossing the STO amplitude threshold set at 5.5 mV for both 3×3 and 5×5 networks. Bar plots show mean value ± SEM and n = 250 for both network sizes. Time until the 5.5 mV STO amplitude threshold is crossed for 3×3 networks is 241±11 ms on average and 308±10 ms on average for 5×5 networks (left panel). The mean p [file pcbi.1002814.s001.pdf]
