# Supplementary material for: Climbing Fiber Burst Size and Olivary Sub-threshold Oscillations in a Network Setting
Source: PLoS Comput Biol. 2012 Dec 13;8(12):e1002814. doi: 10.1371/journal.pcbi.1002814 (PMC3521668; doi:10.1371/journal.pcbi.1002814)
Supplement: Text S1 — This supplementary text contains a full description of the dendritic, somatic and axon hillock compartments of the computational model used. (PDF) [file pcbi.1002814.s002.pdf]

Text S1

The model we used for the simulations described in the paper was based on a two-compartmental model by Schweighofer et al. [1] with a revised gap junction dynamics function [2], a dendritic rather than somatic h current [3], the addition of an axon hillock compartment (this paper) and a reworking of sodium and somatic potassium currents (this paper). The axon hillock sodium current's inactivation function was altered to allow for the generation of bursts of spikes. The functions used to describe the currents are specified in Tables S1-S3 below.

Table S1 - Somatic components

	Current	Activation	Inactivation
**Low-threshold calcium**<br>[1]	$I_{CaL} = G_{CaL} \cdot k^3 \cdot l \cdot (V - V_{Ca})$<br>$G_{CaL} = 0.7\ mS/cm^2$ (default)<br>$0.55\ mS/cm^2 \le G_{CaL} \le 0.9\ mS/cm^2$ (range)	$k_\infty = \frac{1}{1 + e^{{-V-61}/{4.2}}}$<br>$\tau_k = 1$	$l_\infty = \frac{1}{1 + e^{{V+85.5}/{8.5}}}$<br>$\tau_l = \frac{20e^{{V+160}/{30}}}{1 + e^{{V+84}/{7.3}}} + 35$
**Sodium**<br>[4]	$I_{Na} = G_{Na} \cdot {m_\infty}^3 \cdot h \cdot (V - V_{Na})$<br>$G_{Na} = 120\ mS/cm^2$	$m_\infty = \frac{1}{1 + e^{{-V-30}/{5.5}}}$	$h_\infty = \frac{1}{1 + e^{{-V-70}/{-5.8}}}$<br>$\tau_h = 3e^{\frac{-V-40}{33}}$
**Potassium, slow component**<br>[5],[6]	$I_{Kdr} = G_{Kdr} \cdot n \cdot p \cdot (V - V_K)$<br>$G_{Kdr} = 9\ mS/cm^2$	$n_\infty = \frac{1}{1 + e^{{-V-3}/{10}}}$	$p_\infty = \frac{1}{1 + e^{{-V-51}/{-12}}}$
		$\tau_n = \tau_p = 47e^{\frac{-V-50}{900}} + 5$	
**Potassium, fast component**<br>[7]	$I_K = G_K \cdot x^4 \cdot (V - V_K)$<br>$G_K = 5\ mS/cm^2$	$\alpha_x = \frac{0.13V + 3.25}{1 - e^{{-V+25}/{10}}}$<br>$\beta_x = 1.69e^{-0.0125V - 0.4375}$<br>$x_\infty = \frac{\alpha_x}{\alpha_x + \beta_x}$<br>$\tau_x = \frac{1}{\alpha_x + \beta_x}$	

Table S2 - Axon hillock components

	Current	Activation	Inactivation
**Sodium** (adapted from: [4])	$I_{Na} = G_{Na} \cdot {m_\infty}^3 \cdot h \cdot (V - V_{Na})$ $G_{Na} = 240\, mS/cm^2$	$m_\infty = \frac{1}{1 + e^{-V - 30}/_{5.5}}$	$h_\infty = \frac{1}{1 + e^{-V - 60}/_{-5.8}}$ $\tau_h = 1.5e^{\frac{-V - 40}{33}}$
**Potassium** [7]	$I_K = G_K \cdot x^4 \cdot (V - V_K)$ $G_K = 20\, mS/cm^2$	$\alpha_x = \frac{0.13V + 3.25}{1 - e^{-V + 25}/_{10}}$ $\beta_x = 1.69e^{-0.0125V - 0.4375}$ $x_\infty = \frac{\alpha_x}{\alpha_x + \beta_x}$ $\tau_x = \frac{1}{\alpha_x + \beta_x}$	

Table S3 - Dendritic components

	Current	Activation
**High-threshold calcium** [1]	$I_{CaH} = G_{CaH} \cdot r^2 \cdot (V - V_{Ca})$ $G_{CaH} = 4.5\, mS/cm^2$ Dendritic calcium concentration: $\frac{\partial [Ca^{2+}]}{\partial t} = -3I_{CaH} - 0.075[Ca^{2+}]$	$\alpha_r = \frac{1.7}{1 + e^{-V - 5}/_{13.9}}$ $\beta_r = \frac{0.02V + 0.17}{e^{V + 8.5}/_5 - 1}$ $r_\infty = \frac{\alpha_r}{\alpha_r + \beta_r}$ $\tau_r = \frac{5}{\alpha_r + \beta_r}$
**Calcium-dependent potassium** [1]	$I_{K\_Ca} = G_{K\_Ca} \cdot s \cdot (V - V_K)$ $G_{K\_Ca} = 35\, mS/cm^2$	$\alpha_s = min(\{0.00002[Ca^{2+}] \;\; 0.01\})$ $\beta_s = 0.015$ $s_\infty = \frac{\alpha_s}{\alpha_s + \beta_s}$ $\tau_s = \frac{1}{\alpha_s + \beta_s}$

**h current** [3]	$I_h = G_h \cdot q \cdot (V - V_h)$ $G_h = 0.15\ mS/cm^2$	$q_\infty = \frac{1}{1 + e^{V + 80/4}}$ $\tau_q = \frac{1}{e^{-0.086V - 14.6}} + e^{0.07V - 1.87}$

In addition, every compartment has a passive leak current:

$$I_{leak} = G_{leak} \cdot (V - V_{leak})$$

$$G_{leak} = 0.016\ mS/cm^2$$

Similarly, interaction between compartments is also modeled passively, but takes the surface ratio between compartments into consideration additionally:

$$I_{interact} = \frac{G_{internal}}{p_{a,b}} \cdot (V_a - V_b)$$

$$G_{internal} = 0.13\ mS/cm^2$$

Surface ratios: dendrite:soma = 4:1
soma:axon hillock = 20:3

The reversal potentials that were used are as follows:

Reversal potentials (mV)
$V_{Na} = 55$ $V_{Ca} = 120$ $V_K = -75$ $V_h = -43$ $V_{leak} = 10$

For modeling the non-linearity of gap junctional coupling between dendritic compartments of cells, the following function from Schweighofer et al. [2] to modify the coupling conductance (set at a maximum of 0.04 throughout all simulations):

$$w = 0.8 \cdot e^{-V^2/100} + 0.2$$

In the function above, *V* denotes the difference in dendritic membrane potentials between coupled cells.

Cells were organized in a grid and connected to up to eight directly neighboring cells without any thoroidal connections occurring. Because of this, cells at the corner of the grid would only be connected directly to three other cells, cells bordering on the edge of the grid would be connected to five other cells and all other cells to eight neighboring cells. Indirectly, all cells were connected.

Text S1 references

1. Schweighofer N, Doya K, Kawato M (1999) Electrophysiological properties of inferior olive neurons: A compartmental model. J Neurophysiol 82: 804–817.

2. Schweighofer N, Doya K, Fukai H, Chiron JV, Furukawa T, et al. (2004) Chaos may enhance information transmission in the inferior olive. Proc Natl Acad Sci U S A 101: 4655–4660.

3. Van Der Giessen RS, Koekkoek SK, van Dorp S, De Gruijl JR, Cupido A, et al. (2008) Role of olivary electrical coupling in cerebellar motor learning. Neuron 58: 599–612.

4. Parri HR, Crunelli V (1998) Sodium current in rat and cat thalamocortical neurons: Role of a non-inactivating component in tonic and burst firing. J Neurosci 18: 854–867.

5. Bekkers JM (2000) Properties of voltage-gated potassium currents in nucleated patches from large layer 5 pyramidal neurons of the rat. J Physiol 525: 593–609.

6. Korngreen A, Sakmann B (2000) Voltage-gated K^+ channels in layer 5 neocortical pyramidal neurones from young rats: Subtypes and gradients. J Physiol 525: 621–639.

7. D'Angelo E, Nieus T, Maffei A, Armano S, Rossi P, et al. (2001) Theta-frequency bursting and resonance in cerebellar granule cells: experimental evidence and modeling of a slow k+-dependent mechanism. J Neurosci 21: 759–770.
